# Computational Prediction of the Pathogenic Status of Cancer-Specific Somatic Variants

**DOI:** 10.3389/fgene.2021.805656

**Published:** 2022-01-18

**Authors:** Nikta Feizi, Qian Liu, Leigh Murphy, Pingzhao Hu

**Affiliations:** ^1^ Department of Biochemistry and Medical Genetics, University of Manitoba, Winnipeg, MB, Canada; ^2^ Department of Computer Science, University of Manitoba, Winnipeg, MB, Canada; ^3^ CancerCare Manitoba Research Institute, Winnipeg, MB, Canada

**Keywords:** somatic variants, computational classification, pathogenic status, breast cancer, survival analysis

## Abstract

*In-silico* classification of the pathogenic status of somatic variants is shown to be promising in promoting the clinical utilization of genetic tests. Majority of the available classification tools are designed based on the characteristics of germline variants or the combination of germline and somatic variants. Significance of somatic variants in cancer initiation and progression urges for development of classifiers specialized for classifying pathogenic status of cancer somatic variants based on the model trained on cancer somatic variants. We established a gold standard exclusively for cancer somatic single nucleotide variants (SNVs) collected from the catalogue of somatic mutations in cancer. We developed two support vector machine (SVM) classifiers based on genomic features of cancer somatic SNVs located in coding and non-coding regions of the genome, respectively. The SVM classifiers achieved the area under the ROC curve of 0.94 and 0.89 regarding the classification of the pathogenic status of coding and non-coding cancer somatic SNVs, respectively. Our models outperform two well-known classification tools including FATHMM-FX and CScape in classifying both coding and non-coding cancer somatic variants. Furthermore, we applied our models to predict the pathogenic status of somatic variants identified in young breast cancer patients from METABRIC and TCGA-BRCA studies. The results indicated that using the classification threshold of 0.8 our “coding” model predicted 1853 positive SNVs (out of 6,910) from the TCGA-BRCA dataset, and 500 positive SNVs (out of 1882) from the METABRIC dataset. Interestingly, through comparative survival analysis of the positive predictions from our models, we identified a young-specific pathogenic somatic variant with potential for the prognosis of early onset of breast cancer in young women.

## 1 Introduction

Leverage of high-throughput technologies has given rise to an ever-increasing list of sequenced genes, exomes, transcriptomes and genomes. However, availability of a great amount of raw data would not be valuable without being translated into useful information (Trisilowati and DG, 2012). Genomic variants identified through sequencing can relate to susceptibility to complex diseases such as cancer. This is particularly applicable to the variants that affect the genes associated with critical cellular events such as cell cycle process regulation, DNA mismatch repair, metabolism and immunity (Landau et al., 2015; Oldridge et al., 2015). Accurate interpretation of the sequence data helps to choose the most efficient therapy, predict responses to a therapy, and estimate critical clinical consequences such as patient’s overall survival, tumor recurrence-free survival, etc. (Richards et al., 2015).

Our understanding of the pathogenicity of any given genomic variant falls into a spectrum between almost certainly pathogenic to almost certainly benign for a disease (Richards et al., 2015). A principal aim in cancer research has been to identify the mutations affecting the genes with causal roles in cancer susceptibility. Following the report of the first somatic mutation identified in a human oncogene (Reddy et al., 1982; Tabin et al., 1982), a substantial number of oncogenes and their relevant somatic mutations have been detected (Futreal et al., 2004). These mutations can be either pathogenic driver variants, conferring fitness advantages to tumor cells (Hodis et al., 2012), or passenger benign variants, biologically neutral mutations with no growth/survival advantages (Greenman et al., 2007). The biggest challenge of all systemic mutation screenings is to distinguish between the two groups of variants.

In-silico experimentation approaches combine mathematical strategies with expert opinion to interpret the biological significance of genomic data (Trisilowati and DG, 2012) in an efficient and economical manner. In-silico approaches save laboratory costs while allowing for numerous experiments to be conducted simultaneously, be observed and controlled at any level of detail, and be repeated as many times as desired. Many experts in the closely related areas of theoretical and computational biology have shared a view that in-silico experiments can be used as a pioneer or in association with experimental studies (Trisilowati and DG, 2012).

To date, many computational tools have been developed to classify pathogenic status of genomic variants using different training approaches and datasets. Most classification tools have been only benchmarked for classifying the type of variants (e.g. germline variants) included in their original training dataset (Gonzalez-Perez et al., 2013), while being widely used for classifying other types of variants (e.g. both germline and somatic variants) as well. A good example of this circumstance is implementation of FATHMM-MKL (designed based on the characteristics of germline non-cancer variants) for predicting the pathogenic status of cancer somatic mutations in Catalogue of Somatic Mutations in Cancer (COSMIC) dataset.

Considering the importance of both coding and noncoding somatic point mutations in cancer initiation and progression, as well as the increasing emergence of cancer sequence databases such as the international Cancer Genome Consortium (Zhang et al., 2011), The Cancer Genome Atlas (Weinstein et al., 2013), Genomics England (100,000 genomes) Projects (Samuel and Farsides, 2017) there is a strong demand for development of interpretation tools specified for classifying pathogenic status of cancer somatic variants based on the model trained on cancer somatic variants. Here, we developed a computational tool specified for classification of pathogenic status of cancer somatic single nucleotide variants (SNVs). Our models trained on only cancer somatic variants are capable of classifying both coding and noncoding somatic SNVs into two distinct groups of pathogenic somatic mutations and non-pathogenic somatic mutations.

Despite the low occurrence rate of breast cancer in individuals under the age of 40 (7% in developed world and 25% in developing world) compared to their older counterparts, they suffer from more severe presentation, lower survival rate, and higher risks of disease relapse (Saghir et al., 2007; Azim et al., 2012). Various studies have examined patterns of somatic mutations in breast cancer patients. However, there exists a lack of evidence about the landscape of somatic mutations in young patients (Stephens et al., 2012). Using our classification models, we investigated cancer somatic variants in young breast cancer patients to identify the potential age-specific biomarkers and genomic signatures.

## 2 Materials and Methods

**FIGURE 1:**
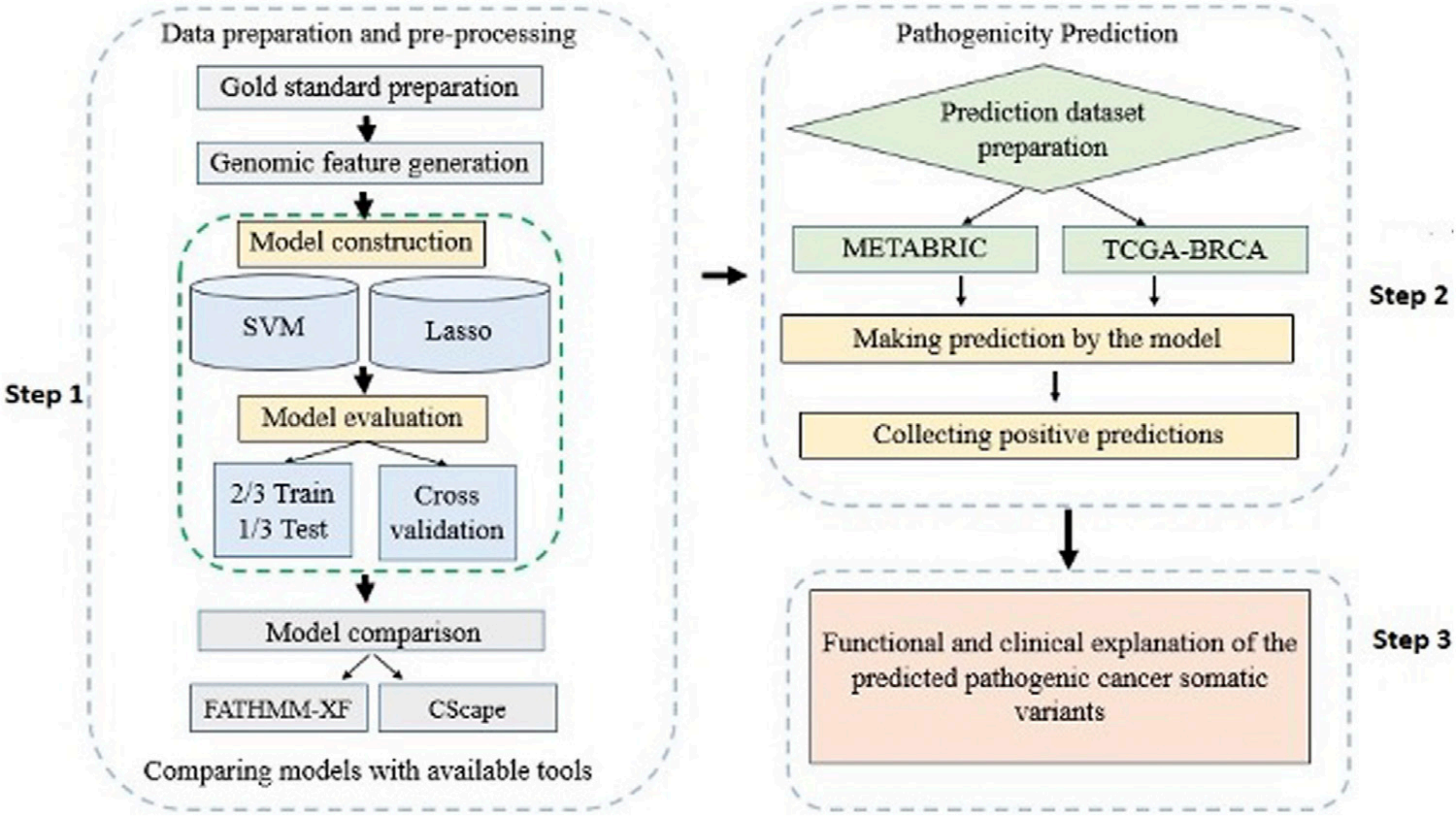
A flowchart overview of the steps of the study modelling and application of computational algorithms designed based on supervised classification methods.

A detailed workflow of the methods and materials used in this study is delineated in Figure 1. The materials include: 1) a list of labeled cancer somatic SNVs constituting the gold standard dataset, 2) a list of cancer un-labeled somatic SNVs constituting the prediction dataset, and 3) feature sets that characterize the cancer somatic SNVs in the gold standard.

### 2.1 Gold Standard

Our gold standard dataset involves somatic SNVs identified in coding and non-coding regions of the DNA from either cancer patients or healthy individuals. Generally, mutations occurring in coding and non-coding regions of the genome share the same basic characteristics such as the innate changes they introduce into the DNA sequence. However, coding mutations can be studied from additional aspects in terms of affected genes, transcripts, and proteins. Accordingly, we split our gold standard into coding and non-coding subsets to train each set individually based on their relevant features. Labels assigned to the pathogenic status of variants in the gold standard form the foundation of model development. Therefore, it is of critical importance that maximum precautions be taken in labeling the pathogenic (positive) and non-pathogenic (negative) examples.

#### 2.1.1 Labelling Positive Examples

Our positive (pathogenic) examples are cancer somatic SNVs extracted from COSMIC database (Forbes et al., 2011) which assembles and organizes thorough information about cancer somatic mutations from majority of known cancer types. To filter out pathogenic SNVs from COSMIC, we first excluded all the SNVs that were mutual between healthy individuals and cancer patients. Next, we defined a recurrence threshold to ascertain that our positive samples are true representatives of pathogenic somatic SNVs. Rogers et al. define a threshold as the number of repetitions of a given mutation across the whole dataset. They based their choice of the best threshold on the size of the remaining positive dataset after filtering out mutations with a frequency of less than a given threshold. In this approach, increasing the threshold decreases the number of remaining positive samples to the point that an extremely high threshold may result in a potential bias by limiting the samples pertaining to only a set of relevant genes. They suggested that the best threshold would provide the classifier with a sufficient number of training examples while introducing the minimum bias (Rogers et al., 2017). In this study, we followed a more conservative approach by defining a bi-dimensional threshold spanning both frequency of a given SNV across the dataset, as well as the number of cancer types in which the mutation occurred. The novelty of this score is that it considers occurrence of a given mutation in more than one cancer type. This is a valid approach as traditionally an obvious step in defining the clinical implications of a new mutation is to determine if it has been involved in other cancers or disorders (Gonzalez-Perez et al., 2013; Rogers et al., 2017).

#### 2.1.2 Labelling Negative Examples

Our negative (non-pathogenic) dataset includes SNVs from COSMIC chosen based on information from dbSNP database (Sherry et al., 2001) and 1,000 Genome Project (Altshuler et al., 2012). Firstly, we extracted the SNVs with a minor allele frequency of equal or greater than 1% in at least one, 1,000 Genomes population reported in dbSNP. This assured us that the collected SNVs are confidently non-pathogenic as reported by two validated resources of genetic variations in healthy individuals. At this point, our negative dataset was a mixture of germline and somatic variants. To extract somatic SNVs from this mixture, we first collected all the SNVs annotated as “found in healthy individuals” from COSMIC, providing us with somatic variants, and subsequently identified the mutual SNVs between these mutations and our mixture. Eventually, we were confident each SNV in our negative dataset is a somatic benign variant found in healthy individuals.

### 2.2 Genomic Features

One goal of our classification models is to learn the discrepancies and similarities between pathogenic and non-pathogenic cancer somatic SNVs. To this aim, we used genomic features characterizing mutations through criteria such as sequence characteristics, genomic content of the mutation sites, and functional and structural consequences of the mutations. Genomic features defined in this study are mostly annotations from different projects such as ENCODE (Dunham et al., 2012), CADD (Rentzsch et al., 2019), and ENSEMBL variant effect predictor (VEP) (McLaren et al., 2016), scores from pathogenic SNV predictors such as POLYPHEN (Adzhubei et al., 2013) and SIFT (Ng and Henikoff, 2003), as well as information from variant browsers such as BRAVO (bravo.sph.umich.edu/freeze5/hg38). We have grouped the features into four major subsets, each portraying the mutations from a specific aspect including 1) structural and genomic context features, 2) epigenetic features, 3) genomic distance features, 4) genomic conservation features, described in Table 1. Overall, we defined 65 genomic features for coding and non-coding variants, with an additional 15 coding specific features adding up to a total of 80 features that were included in coding gold standard (Supplementary Table S1).

**TABLE 1:** Four major genomic feature groups characterizing the SNVs in gold standard datasets.

Feature group	Description	Example
Structural and genomic context features	Characterizing sequence attributes of the mutation location. These features estimate the disruption in the mutations surrounding sequence both in coding and non-coding regions	Percentage of GC in a ±75 bp window
Epigenetic features	Describing epigenetic changes such as histone modifications and methylation alterations	Maximum H3K4 methylation level from Encode
Genomic distance features	Measuring the distance between a given SNV and critical functional and structural genomic elements such as transcription start and end sites	Minimum distance to Transcribed Sequence Start (TSS)
Genomic conservation features	Measuring the evolutionary conservation at the mutation alignment sites in an effort to help the training models learn the relationships between the measurements and pathogenicity of the SNVs	Scores from PhastCons and Phylop

### 2.3 Handling Missing Data

Like most data collection-based studies, missing data were inevitable in our study. Our sample size would shrink significantly (more than 5%) in the event of removing all the SNVs that have any missing values. Therefore, the MICE (Multivariate imputation by chained equations) imputation approach was used for estimating the potential values of the missing data. MICE is applicable to datasets with missing values in multiple variables (Wulff and Jeppesen, 2017), and is also adapted to handle different types of data (e.g. continuous or binary) (Van Buuren and Groothuis-Oudshoorn, 2011).

### 2.4 Data Normalization

Data normalization or scaling is known to be beneficial in improving the performance of some classifiers such as support vector machine (SVM). To investigate the effect of data normalization on our models we used Python Scale package from sklearn library (Pedregosa et al., 2011) to normalize the feature values in our gold standard datasets. Generally, two major issues are addressed in data normalization: first, to avoid large values in wider numeric scales which can cause numerical problems; second, to simplify the classification calculation process.

### 2.5 Classification Methods

In this study we have used two relatively popular classification methods with demonstrated capability of dealing with cancer genomic data:

1) Lasso (least absolute shrinkage and selection operator) regression model is a type of linear regression that sums up penalty scores equal to the absolute value of the coefficients resulting in elimination of features with large penalty scores (Tibshirani, 1996). The final coefficients estimated by Lasso regression indicate the contribution of each feature in predicting the outcome value. Lasso conducts feature selection by setting the coefficients of non-discriminative features to zero. This is especially suitable for models with high levels of multicollinearity (Farrar and Glauber, 1964) which occurs when there is a high correlation between two or more features in the model.
2) SVM is a powerful classification method capable of predicting labels of two classes based on their defined features (Huang et al., 2018). SVM discriminates the two groups by creating a decision boundary called hyperplane which is oriented in a way that keeps the largest possible distance from the closest data points of each class known as support vectors. In addition to linear classification, SVM is supplied with a kernel method which facilitates certain calculations needed for high dimensional space non-linear classification (Huang et al., 2018). Among other parameters, choice of the kernel can affect SVM classification power enormously. However, there is no certain way of choosing the best kernel without conducting trial and error practices starting from a simple SVM model.

### 2.6 Model Evaluation

We used two strategies to evaluate our model’s performance. In the first approach, we used 2/3 of the total samples for training and the remaining 1/3 for testing. In the second approach, we performed 10-fold cross-validation (10F CV) of each of the gold standards. We used area under the ROC (receiver operating characteristic) curve as an estimation of the discrimination power of our classification models (Cook, 2007).

### 2.7 Applying the Models to Breast Cancer Cohort Studies

We studied the landscape of somatic SNVs in young breast cancer patients by applying our trained models to the data from two cohort studies including METABRIC (Molecular Taxonomy of Breast Cancer International Consortium) (Pereira et al., 2016) and TCGA-BRCA (The Cancer Genome Atlas Breast Invasive Carcinoma) (Grossman et al., 2016), both representing the genomic profile of breast cancer tumors. In the interest of our study aims, we only examined the SNVs from patients younger than 45 years of age.

METABRIC is based on exome sequencing of 173 previously established risk genes from 2,433 primary breast cancer samples. From a total of 32,476 SNVs identified in METABRIC, we were able to define the genomic features for 13,942 somatic SNVs of which 1882 were from 326 patients younger than 45 years of age at diagnosis. TCGA-BRCA characterizes human breast tumors using five molecular assessment platforms involving Affymetrix SNP arrays, Illumina Infinium DNA methylation chips, Agilent mRNA expression microarrays, whole exome sequencing and microRNA sequencing. From a total of 80,227 somatic SNVs from 976 patients, we were able to define the genomic features for 8,647 somatic SNVs from 142 young patients (<45 years old at diagnosis). Regarding the data collection approaches followed by TCGA-BRCA study, 6,910 somatic SNVs were from coding regions (e.g., provided by whole exome sequencing) and 1737 somatic SNVs were from noncoding regions (e.g. provided by microRNA sequencing) of the genome.

#### 2.7.1 Survival Analysis

Based on different events of interests we conducted two types of survival analysis in our study.

1) SNV-level survival analysis, which regards the recurrence of a given mutation in a given patient as the event of interest, was conducted for the somatic SNVs from METABRIC and TCGA-BRCA that were predicted to be pathogenic by our models. We were particularly interested in the predicted somatic pathogenic mutations with the most frequency (recurrence frequency of ≥4 and ≥2 in coding and noncoding regions, respectively). SNV-level survival analysis assesses whether the survival time of the patients who harbor a specific mutation in their tumour genome is significantly different from the patients whose tumours do not harbor the mutation. We identified the mutations that were significantly associated with the survival time of young patients and compared the results with outcomes from SNV-level survival analysis of old patients.
2) Gene-level survival analysis, when considering a given gene as mutated or not as the event of interest, was conducted for the genes from METABRIC and TCGA-BRCA data sets. Consistent with the purpose of our study we were only interested in those genes whose mutation status was significantly associated with the survival time of young patients but not the survival time of the older patients. We defined a gene as mutated if it was affected by at least one predicted pathogenic SNV in a given sample.

#### 2.7.2 Gene Set Enrichment Analysis (GSEA)

Gene set enrichment analysis also referred to as functional enrichment analysis is an analytical method that determines whether the members of a given list of genes are over-represented in an *a priori* known set of genes or proteins (Subramanian et al., 2005). GSEA also helps in investigating the association between expression of a given list of genes with disease phenotypes. In this study we used Enrichr software (Chen et al., 2013; Kuleshov et al., 2016) to conduct enrichment analysis investigating whether the genes that are affected by the somatic SNVs predicted as pathogenic by our models are over-represented in any interesting cellular pathway or function.

## 3 Results

### 3.1 Gold Standard

Following our bi-dimensional scoring approach for labeling positive cancer somatic SNVs, we computed the number of SNVs reaching different thresholds of each dimension of the scoring methods shown in Supplementary Table S2 and Supplementary Table S3 for coding and non-coding datasets, respectively. For example, the highlighted cells in Supplementary Table S2 indicate 122,054 SNVs are repeated at least 4 times across the whole dataset, and 206,349 SNVs are identified in at least two cancer types (e.g. skin and breast cancers). Following the “sufficient examples with minimum bias” condition, in coding dataset, four and two were chosen as optimal thresholds regarding the first and second dimension of the final score, respectively. The chosen threshold for the first and second dimensions of the final score in the non-coding dataset were 3 and 2, respectively. Ultimately, the coding gold standard included 12,313 positive (pathogenic) and 16,594 negative (non-pathogenic) cancer somatic variants. and the non-coding gold standard included 28,993 positive (pathogenic) and 58,995, negative (non-pathogenic) SNVs, respectively.

### 3.2 Most Discriminative Genomic Features

Identifying the features with highest contribution to the discrimination power of a model is important in evaluating the model from biological aspects. In the Lasso model the bigger the absolute value of the coefficient of a feature the more discriminative the feature. Accordingly, the coefficient of zero effectively implies that the feature is discarded in the feature selection process and has not been used for model training. The coefficients of features from coding and noncoding Lasso models (Supplementary Table S4 and Supplementary Table S5) indicate that among coding features “structural and genomic context features” (e.g. percentage of CpG islands, GC percentage in a ±75 window from a given mutation, and the number of single occurrence of SNVs (MAF<0.05) in a ±100 window from the given mutation), “genomic distance features” (e.g. relative distance of a SNV from transcription start site), and “genomic conservation features” (e.g. scores form PhastCons, and methylation modifications to protein histone H3 (H3K9me3) at ninth lysine residue) are ranked as the most discriminative features, respectively. Two features including the number of amino acid distance from coding start site and the *p*-values from GerpRS evolution scoring tool have not been used in the coding model as they have a coefficient of 0.

A similar trend was repeated in ranking the most discriminative noncoding features; “Structural and genomic context” (e.g. CpG islands in a ± 75 window from the mutation, number of single occurrences of the SNVs (MAF<0.05) in ± 100 window from the mutation, transverison/transition identity of the nucleotide change, and GC percentage in a ± 75 window from the mutation), “genomic conservation features” (e.g. scores from Phylop), and “epigenetic features” (e.g. methylation modifications to protein histone H3 (H3K36me3) at 36th lysine residue). Interestingly, unlike the coding model, no features had the coefficient of zero in the non-coding Lasso model. Hence, For the Lasso models, 78 and 65 features were used in the models for coding and non-coding regions, respectively. However, for the SVM models, no features were filtered out, and 80 and 65 features were used in the final models for the coding and non-coding regions, respectively.

### 3.3 Model Selection and Visualization

**FIGURE 2:**
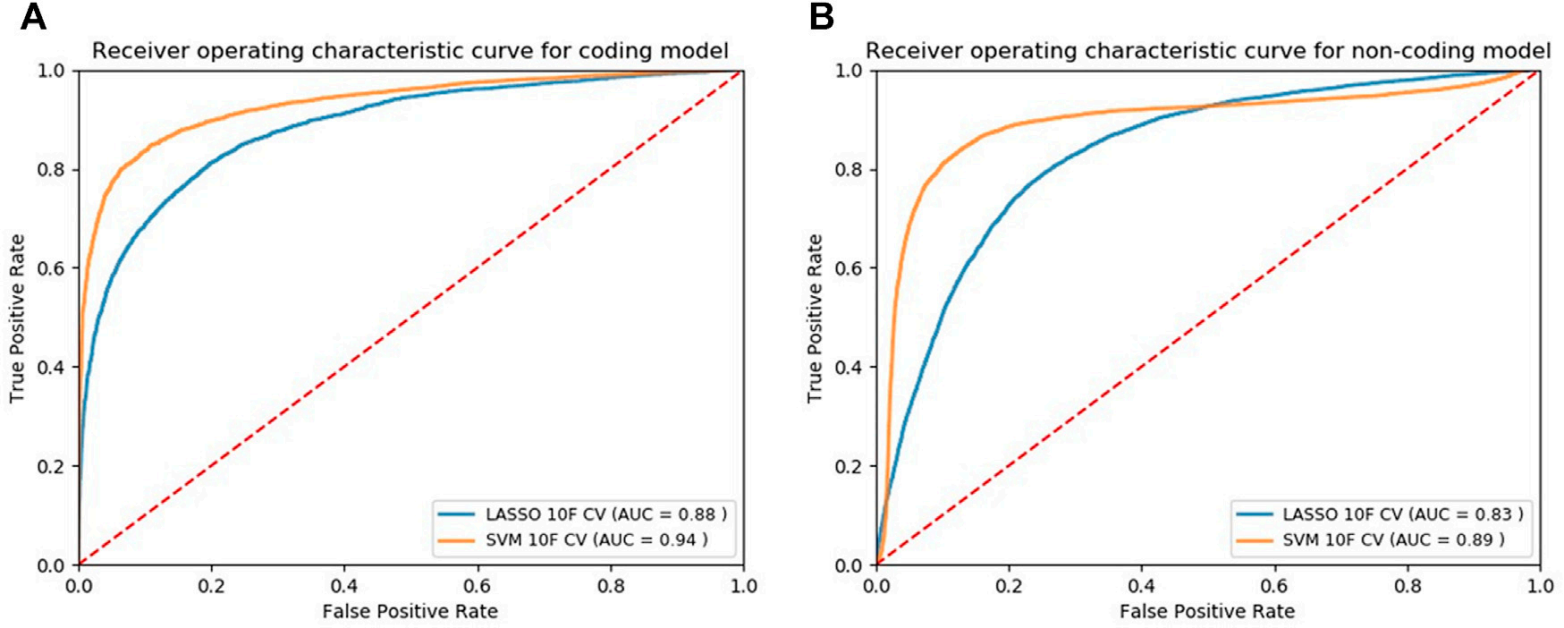
ROC curves of the models designed for classifying cancer somatic variants from coding **(A)** and non-coding **(B)** regions of the genome.

We applied our modeling methods [LASSO and SVM with radial basis function (SVM-rbf)] to both normalized and non-normalized data and evaluated their performance (Supplementary Table S6 and Supplementary Table S7). Using the 10F CV, we observed that data normalization does not affect the performance of Lasso models while significantly increases the classification performance of SVM models. Using the normalized data, we plotted the ROC curves based on 10F CV of the final candidate Lasso and SVM models shown in Figure 2A and Figure 2B for the coding and noncoding models, respectively. Classification models return a continuous probability value (from 0 to 1) which needs to be mapped to a binary category (e.g. pathogenic or non-pathogenic) using a classification threshold. ROC curves can be beneficial in identifying the best classification threshold (also called optimum threshold) yielding the highest true positive and lowest false positive results. We chose the classification threshold of 0.55 as the optimum threshold for our coding model, reaching the true positive rate (TPR) of 0.80 and false positive rate (FPR) of 0.06 (Supplementary Table S8). To acquire the same TPR by our noncoding model we chose the optimum threshold of 0.41, reaching the TPR of 0.80 and FPR of 0.09 (Supplementary Table S9).

### 3.4 Model Benchmarking

**FIGURE 3:**
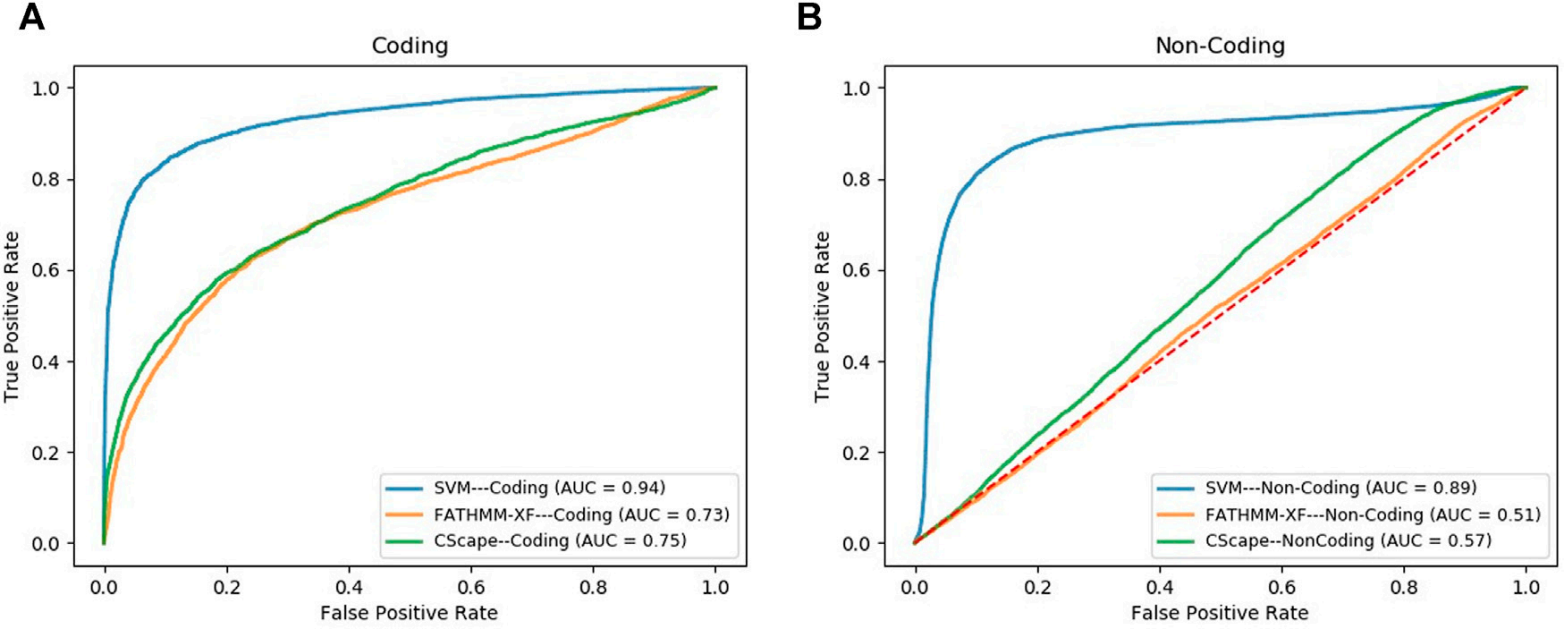
ROC curves comparing the performance of our model (SVM) with FATHMM-XF and CScape for somatic cancer variants in coding **(A)** and non-coding **(B)** regions of the genome.

We compared the performance of our final SVM models, in the task of classifying cancer somatic variants, with two of the leading variant classifiers including FATHMM-XF and Cscape. Using FATHMM web server (http://fathmm.biocompute.org.uk/), we applied FATHMM-XF and Cscape to the same coding and noncoding test data sets used for evaluating our SVM models. Please note that we did not use 10F CV for the model benchmarking, instead, we used the same test data sets used in the train/test strategy for evaluating and comparing the model performance (the details are descripted in the first approach of Section 2.6). Figure 3A and Figure 3B show the ROC curves and the AUC values of the three classifiers for coding and noncoding SNVs, respectively. As evident by the AUC values, SVM models outperformed their competitors in both coding and noncoding datasets. In the task of classifying cancer somatic SNVs, our study suggests the following hierarchy in ranking classifiers based on their performance; 1) A models designed based on the characteristics of only cancer somatic variants (our SVM model), 2) A model designed based on the characteristics of a mixture of somatic and germline cancer variants (Cscape), 3) A model designed based on the characteristics of germline variants (FATHMM-XF).

### 3.5 Model Predictions

Results from applying our SVM models to the prediction datasets (METABRIC, TCGA-coding, TCGA-noncoding) are shown in Table 2. Please note that some somatic pathogenic SNVs used in training set were also predicted to be somatic pathogenic SNVs in the METABRIC and TCGA-BRCA datasets. The second column of Table 2 indicates the number of predicted somatic pathogenic SNVs that are overlapped with the training pathogenic somatic SNVs. Furthermore, there are 21 overlapped somatic pathogenic SNVs between the TCGA and METABRIC cohorts, which are listed in Supplementary Table S10. We reported the number of SNVs predicted as pathogenic as well as the number of genes affected by these SNVs in Supplementary Table S8 and Supplementary Table S9.

**TABLE 2:** Number of pathogenic SNVs. They were predicted by the SVM models for the SNVs from prediction datasets regarding the optimum cut-offs [0.55 for METABRIC and TCGA-coding (TCGA-CD), and 0.41 for TCGA-noncoding (TCGA-NC)].

Dataset	No. of pathogenic predictions	No. of pathogenic predictions overlapped with training SNVs	No. of affected genes	The frequency of pathogenic SNVs ≥2		The frequency of pathogenic SNVs ≥3		The frequency of pathogenic SNVs ≥4	
				No. of SNVs	No. affected of genes	No. of SNVs	No. of affected genes	No. of SNVs	No. of affected genes
METABRIC	959	27	154	52	18	17	5	**12** ^a^	3
TCGA-CD	3,510	59	2,537	232	184	6	2	**4**	2
TCGA-NC	943	4	331	**58**	25	0	0	0	0

a Bold ones are the number of SNVs that were used in the SNV-level survival analysis.

We further investigated the biological aspects of prediction results by identifying the genes that were affected by the most frequent pathogenic SNVs (recurrence frequency of ≥4 and ≥ 2 in coding and noncoding prediction data sets, respectively) predicted by our models. As indicated in Table 3 the genes from coding prediction data sets (METABRIC and TCGA-coding) included AKT1, PIK3CA, and TP53 which all are demonstrated to play a critical role in breast cancer initiation and progression. The genes affected by the pathogenic SNVs from noncoding prediction dataset (TCGA-coding) mostly belong to three major categories including pseudogenes (KRTAP19-11P, AL034345.1, PGAM1P6, CDC27P1), RNA genes (AC211476.2, AC120498.10, RF00092, MIR519A2, AL049555.1), and members of zinc finger gene family (ZNF512, ZFP30, ZDHHC11B).

**TABLE 3:** An overview of the genes harboring the recurrent pathogenic SNVs predicted by our models. The “SNV ID” column shows the ID of the recurrent SNV that affects the gene mentioned in “Gene” column. “Ref” column shows the nucleotide in the reference genome sequence and “Alt” column shows the alternative nucleotide that is substituted for the reference nucleotide. The highlighted row shows the SNV that appeared as significant through our subsequent survival analysis.

Cohort	Gene	SNV ID	Ref	Alt	SNV position [Chr: Position (base pair: GRCh38)]	SNV consequence from VEP Ensembl
METABRIC	AKT1	14:104780214_C > T	C	T	14:104780214	Missense
METABRIC	PIK3CA	**3:179203765_T > A** ^a^	T	A	3:179203765	Missense
METABRIC	PIK3CA	**3:179218294_G > A**	G	A	3:179218294	Missense
METABRIC	PIK3CA	**3:179218303_G > A**	G	A	3:179218303	Missense
METABRIC	PIK3CA	**3:179234297_A > T**	A	T	3:179234297	Missense
METABRIC	TP53	17:7673802_C > T	C	T	17:7673802	Missense
METABRIC	TP53	17:7674220_C > T	C	T	17:7674220	Missense
METABRIC	TP53	17:7674221_G > A	G	A	17:7674221	Missense
METABRIC	TP53	17:7675088_C > T	C	T	17:7675088	Missense
TCGA-CD	PIK3CA	**3:179203765_T > A**	T	A	3:179203765	Missense
TCGA-CD	PIK3CA	**3:179218294_G > A**	T	A	3:179218294	Missense
TCGA-CD	PIK3CA	**3:179218303_G > A**	G	A	3:179218303	Missense
TCGA-CD	TP53	17:7675088_C > T	C	T	17:7675088	Missense
TCGA-NC	ZFP30	19:37613150_G > A	G	A	19:37613150	Missense
TCGA-NC	CLIC3	9:136993900_A > C	A	C	9:136993900	Regulatory_region_SNV
TCGA-NC	AC211476.2	7:72926895_G > C	G	C	7:72926895	Missense
TCGA-NC	ZNF512	2:27578227_C > T	C	T	2:27578227	Missense
TCGA-NC	KRTAP19-11P	21:30541689_G > A	G	A	21:30541689	Missense
TCGA-NC	AL034345.1	6:38924007_C > G	C	G	6:38924007	Missense
TCGA-NC	PGAM1P6	2:23869699_C > A	C	A	2:23869699	Missense
TCGA-NC	ZDHHC11B	5:711218_G > C	G	C	5:711218	Noncoding_exon_SNV
TCGA-NC	AC120498.10	16:1220974_G > A	G	A	16:1220974	Missense
TCGA-NC	RF00092	1:37880149_C > G	C	G	1:37880149	Missense
TCGA-NC	MIR519A2	19:53761153_G > A	G	A	19:53761153	Mature miRNA variant
TCGA-NC	AL049555.1	6:54941625_C > T	C	T	6:54941625	Missense
TCGA-NC	PLIN5	19:4538646_C > T	C	T	19:4538646	Missense
TCGA-NC	CDC27P1	2:132257729_T > G	T	G	2:132257729	Noncoding_exon_SNV

a Bold ones are the SNVs that are overlapped with the somatic pathogenic SNVs in the training set. In other words, they are known somatic pathogenic SNVs.

#### 3.5.1 SNV-Level Survival Analysis

**FIGURE 4:**
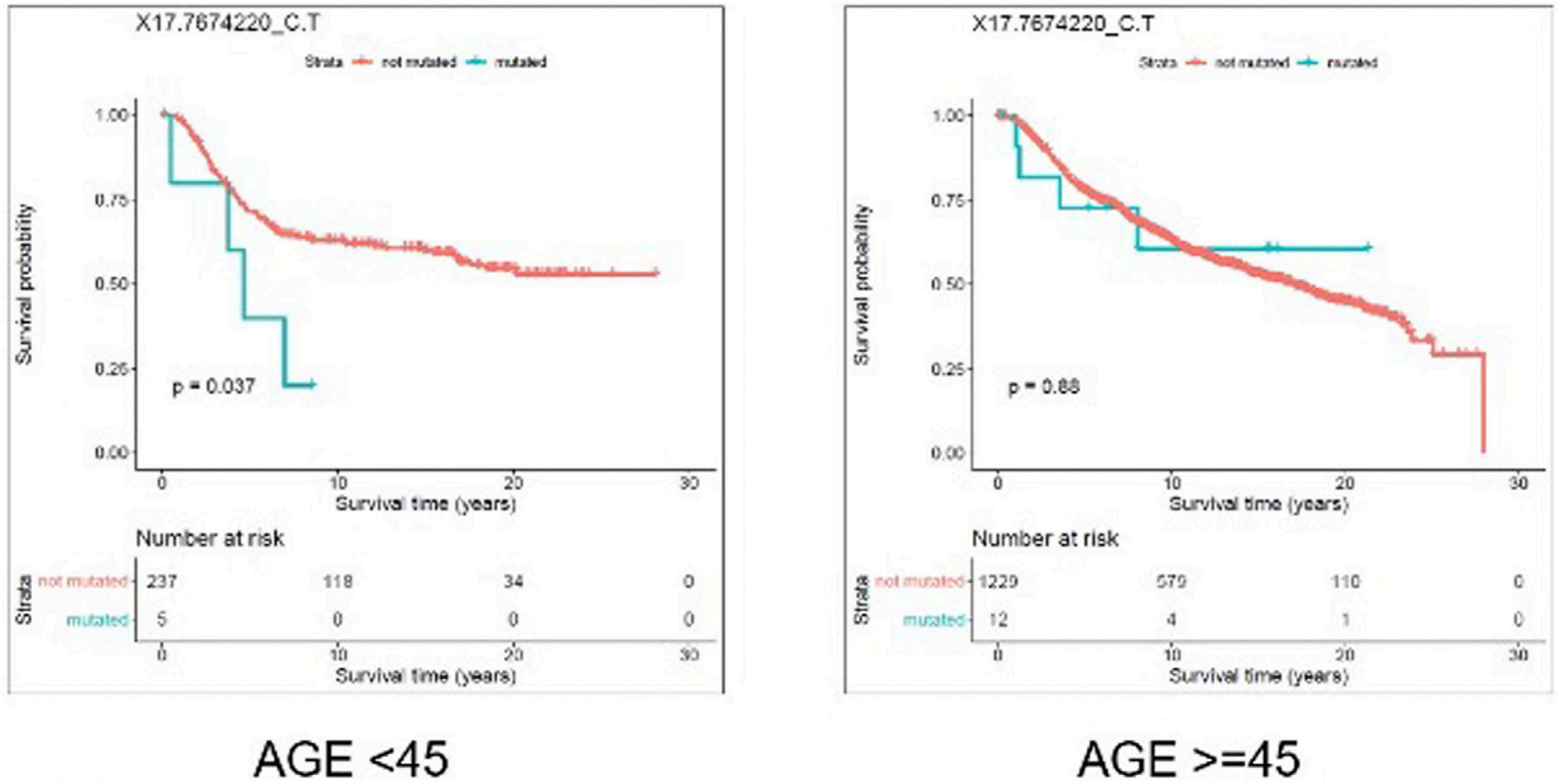
Results from disease specific survival (DSS) analysis comparing the survival time of breast cancer patients with and without the mutation X17.7674220_C.T. The difference between the two groups of patients (with and without the mutation) is significant among young (under 45 years of age) individuals (*p*-value = 0.037), but not significant in older patients (*p*-value = 0.88).

**FIGURE 5:**
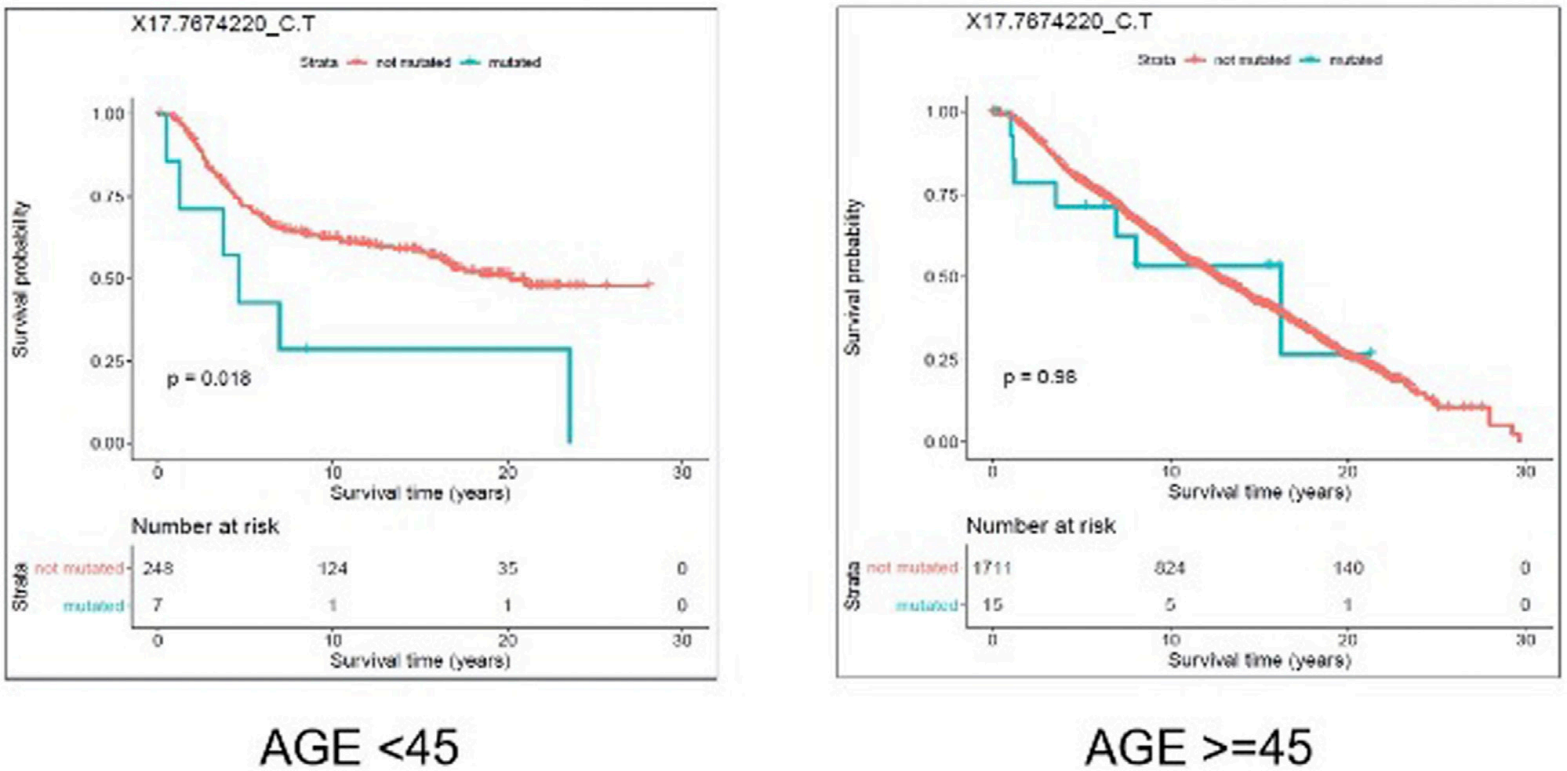
Results from overall survival (OS) analysis comparing the survival time of breast cancer patients with and without the mutation X17.7674220_C.T. The difference between the two groups of patients (with and without the mutation) is significant among young (under 45 years of age) individuals (*p*-value = 0.018), but not significant in older patients (*p*-value = 0.98).

We evaluated the association between predicted pathogenic SNVs with the most frequency (recurrence frequency of ≥4 and ≥2 in coding and noncoding prediction data sets, respectively), and survival outcome of the patients. We grouped the patients based on their age (Age <45 and Age ≥ 45) and compared the survival outcomes between the two groups. The results from disease specific survival analysis (Figure 4) and overall survival analysis (Figure 5) consistently suggested that occurrence of “17:7674220_C > T” SNV is significantly (*p*-value<0.05) associated with the survival experience of patients younger than 45 years of age, while not significantly associated with survival of older patients in the METABRIC data set. However, we did not replicate this finding in the TCGA-Breast data set, which may be due to the small sample size of the young women with breast cancer in the cohort. Following our subsequent investigations, it was found that “17:7674220_C > T” SNV, which is regarded as a “hotspot mutation” in the literature, is an arginine to glutamine single nucleotide substitution occurring at 248th residue of TP53 (Shajani-Yi et al., 2018). We also explored the location of the identified mutation “17:7674220_C > T” (R248) in the three-dimentional structure of TP53 protein using the web service Swiss-PO (Krebs et al., 2021). The structure is shown in Supplementary Figure S1. It is suggested that the mutation disrupts the tumor suppressive activity of TP53 by hindering the binding of the TP53 product to DNA (Bullock and Fersht, 2001; Saha et al., 2015; Soussi and Wiman, 2015).

Furthermore, we investigated the biochemical properties of arginine and glutamine to unravel the impact of their substitution on proteins structure and function. The positively charged guanidinium group in arginine makes it a hydrophilic amino-acid appropriate for being located on the surface of proteins in an aqueous environment (Borders et al., 1994). Arginine has an important role in binding of a protein’s active site to negatively charged cofactors, effectors, substrates (Riordan et al., 1977). Arginine also participates in formation of salt bridges which stabilize the tertiary and quaternary structure of proteins (Borders et al., 1994). Glutamine on the other hand is a polar neutral amino acid. Accordingly, glutamine would likely not compensate for the role arginine would have in maintaining a protein’s structure and function.

#### 3.5.2 Gene-Level Survival Analysis

To select the candidate genes for conducting gene-level survival analysis we computed the number of positive SNVs affecting each gene in the prediction datasets. To be consistent with the frequency threshold we defined for labeling positive examples in our gold standards at the very first steps of the model building process, we selected the genes harboring at least four and two pathogenic SNVs in coding (METABRIC and TCGA-BRCA coding) and noncoding (TCGA-BRCA) datasets, respectively. As inferred from Table 4 the selected thresholds provided us with 55 genes from METABRIC, 29 genes from TCGA-coding, and 22 genes from TCGA non-coding datasets.

**TABLE 4:** Number of genes affected per different thresholds. The thresholds indicate the number of positive somatic point mutations each gene is harboring. The highlighted ones were used in the gene level survial analysis.

Frequency threshold	1	2	3	4	5	6	7	8	9
Count of genes-METABRIC	154	106	74	55	44	36	29	27	23
Count of genes-TCGA-coding	2,539	412	92	29	11	7	3	2	2
Count of genes-TCGA- non-coding	330	22	3	0	0	0	0	0	0

Through gene-level survival analysis of the selected genes we investigated whether the fact that a given gene is mutated or not, can significantly affect the survival experience of the patients. We conducted the gene-level survival analysis for both young (<45 years old) and old (≥45 years old) groups of patients and compared the results from the two groups. The results revealed that mutated “Muc16” gene significantly (*p*-value < 0.05) affected the survival experience of the young patients but did not have a significant (*p*-value > 0.05) impact on the survival of the older patients.

**FIGURE 6:**
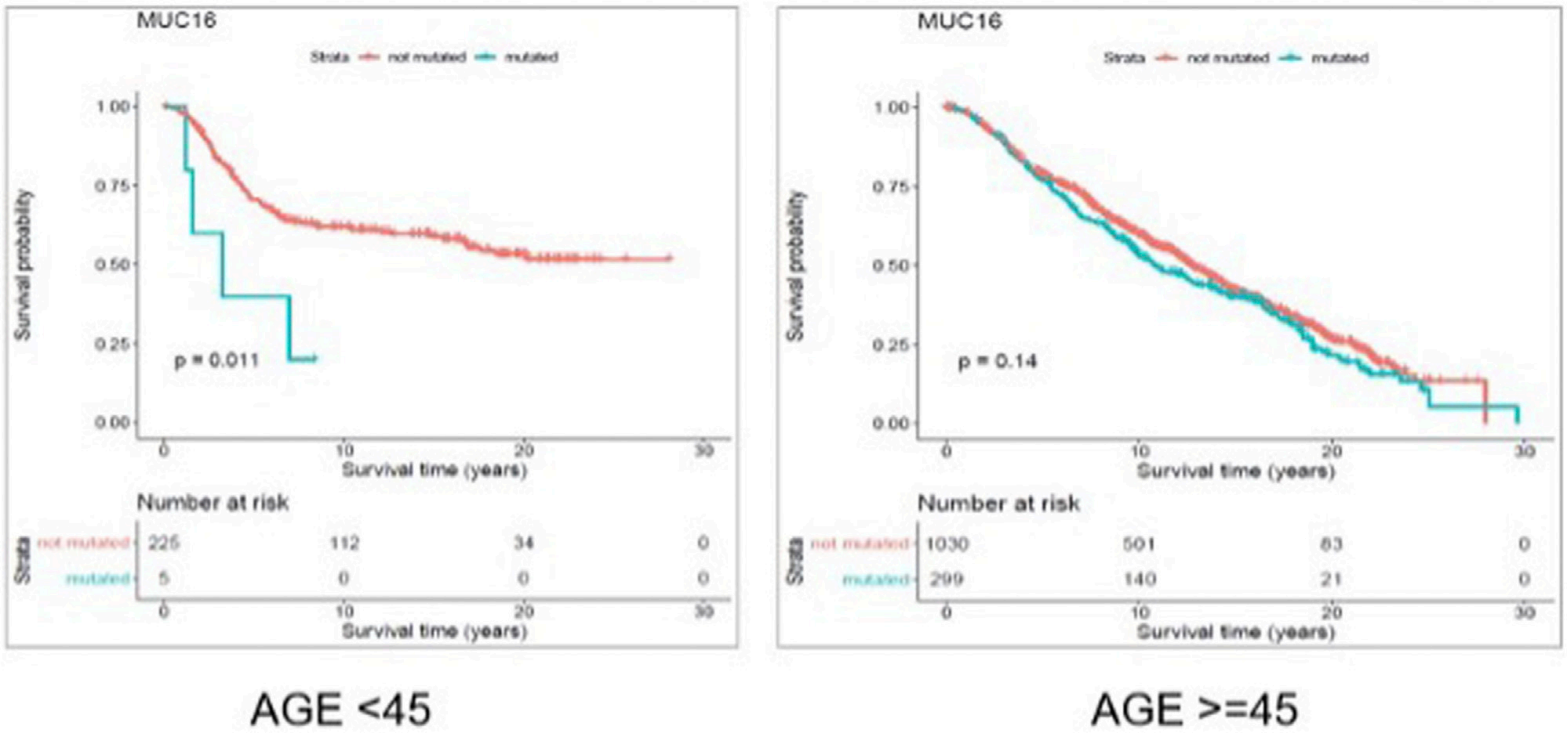
Results from disease free survival (DFS) analysis comparing the survival time of breast cancer patients possessing a mutated or un-mutated Muc16 gene. The difference between the two groups (with and without mutations in Muc16 gene) is significant among young (under 45 years of age) individuals (*p*-value = 0.011), while not significant in older patients (*p*-value = 0.14).

**FIGURE 7:**
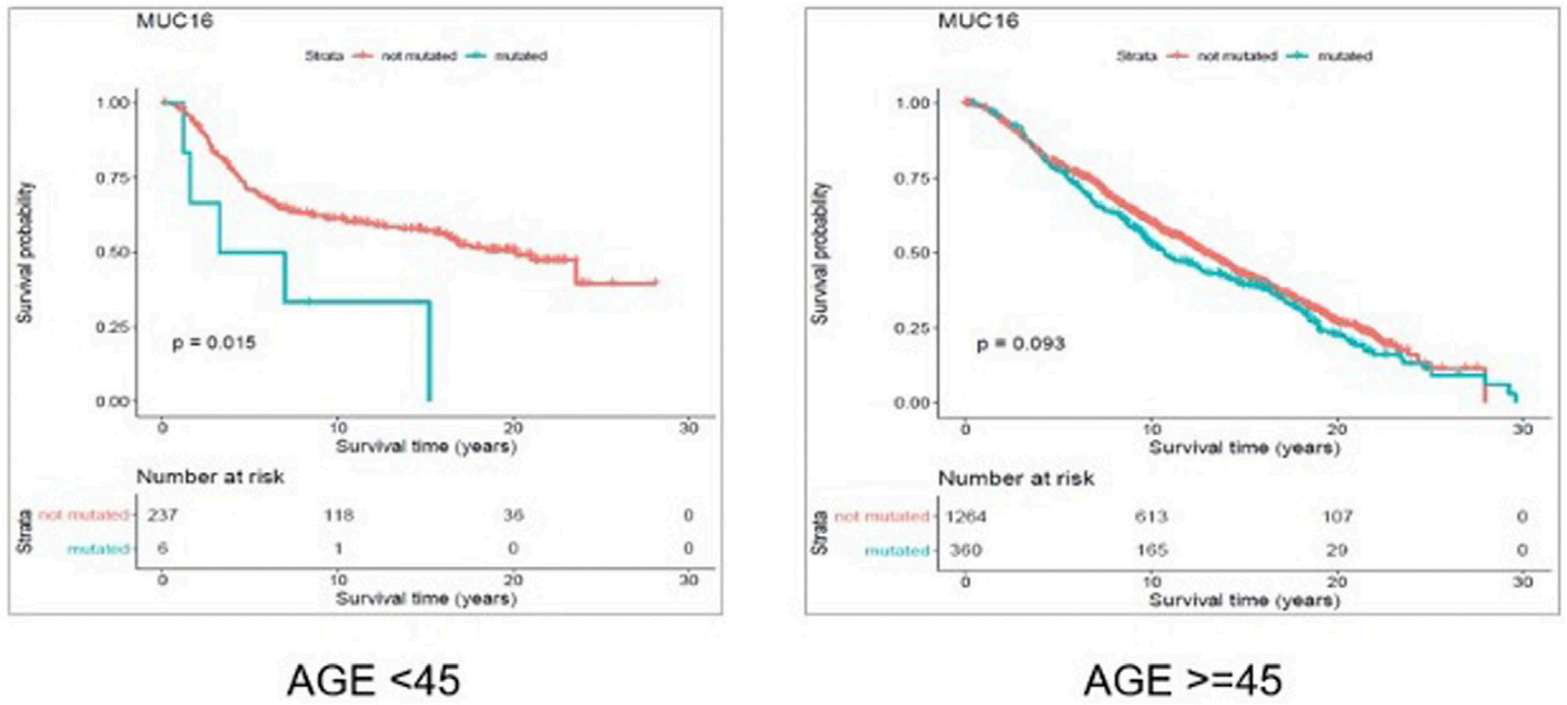
Results from overall survival (OS) analysis comparing the survival time of breast cancer patients possessing a mutated or un-mutated Muc16 gene. The difference between the two groups (with and without mutations in Muc16 gene) is significant among young (under 45 years of age) individuals (*p*-value = 0.011), while not significant in older patients (*p*-value = 0.14).

Figure 6 and Figure 7 show the Kaplan Maier survival plots from disease free and overall survival analysis for “Muc16” gene in the METABRIC data set, respectively. Muc16 expression is demonstrated to be associated with the development of different cancer types including pancreatic (Wu et al., 2009) and breast cancers (Moritani et al., 2008). Lakshmanan et al. suggested that Muc16 contributes to breast cancer progression by increasing cell’s proliferation through interaction with Janus kinase (JAK2) as well as inhibiting cell apoptosis by downregulating of TRAIL (LeBlanc and Ashkenazi, 2003; Lakshmanan et al., 2012). Interestingly, consistent with our findings Muc16 overexpression in epithelial breast cancer tissues is demonstrated to be positively associated with the stage of the disease (Lakshmanan et al., 2012). In addition, Norum et al. regarded the elevated expression of Muc16 in breast cancer as a sign of advanced disease. They demonstrated that increased expression of Muc16 is associated with metastasis and poor prognosis in the stage IV of the disease (Norum et al., 2001).

To our knowledge, to date there is no evidence concerning the association between Muc16 deregulation and early onset of breast cancer. However, association between the stage of breast cancer and elevated Muc16 expression as well as incidence of higher stages of the disease in younger patients support our findings.

#### 3.5.3 Gene Set Enrichment Analysis

We conducted GSEA of the genes affected by the pathogenic SNVs from both coding (METABRIC and TCGA coding) and noncoding (TCGA noncoding) prediction datasets. No significant results (adjusted *p*-value > 0.05) were obtained for the genes from TCGA noncoding dataset. The results of GSEA for the genes from coding datasets are represented in Table 5.

**TABLE 5:** Significant (adjusted *p*-value<0.05) gene sets showing an overrepresentation of our candidate gene lists. For each library we have only reported the five top significant gene sets.

Gene list	Reactome 2016	Panther 2016	KEGG 2019 human	GO biological process 2018	GO molecular function 2018	ChEA 2016
A	Chromatin modifying enzymes_*Homo sapiens*_R-HSA-3247509	EGF receptor signaling pathway_*Homo sapiens*_P00018	Endometrial cancer	positive regulation of nucleic acid-templated transcription	Protein kinase activity	AR_22383394_ChIP-Seq_PROSTATE_CANCER_Human
A	Chromatin organization_*Homo sapiens*_R-HSA-4839726	p53 pathway feedback loops 2_*Homo sapiens*_P04398	Hepatocellular carcinoma	Positive regulation of gene expression	Protein kinase binding	STAT3_23295773_ChIP-Seq_U87_Human
A	Diseases of signal transduction_*Homo sapiens*_R-HSA-5663202	Angiogenesis_*Homo sapiens*_P00005	Pathways in cancer	Positive regulation of transcription, DNA-templated	Transcription coactivator activity	SMAD4_21799915_ChIP-Seq_A2780_Human
A	PI-3K cascade:FGFR1_*Homo sapiens*_R-HSA-5654689	Insulin/IGF pathway-protein kinase B signaling cascade_*Homo sapiens*_P00033	Human papillomavirus infection	Phosphatidylinositol 3-kinase signaling	Ubiquitin protein ligase binding	ZNF217_24962896_ChIP-Seq_MCF-7_Human
A	PI-3K cascade:FGFR2_*Homo sapiens*_R-HSA-5654695	Apoptosis signaling pathway_*Homo sapiens*_P00006	Breast cancer	Chromatin disassembly	Ubiquitin-like protein ligase binding	DROSHA_22980978_ChIP-Seq_HELA_Human
B	Neuronal System_*Homo sapiens*_R-HSA-112316	Endothelin signaling pathway_*Homo sapiens*_P00019	Endometrial cancer	Calcium ion import	Calcium ion transmembrane transporter activity	STAT3_23295773_ChIP-Seq_U87_Human
B	Transmission across Chemical Synapses_*Homo sapiens*_R-HSA-112315	p53 pathway feedback loops 2_*Homo sapiens*_P04398	PI3K-Akt signaling pathway	Axonogenesis	ATPase activity (	TCF4_23295773_ChIP-Seq_U87_Human
B	PI-3K cascade:FGFR1_*Homo sapiens*_R-HSA-5654689	p53 pathway_*Homo sapiens*_P00059	Pathways in cancer	Calcium ion transmembrane transport	Calcium channel activity	SMAD4_21799915_ChIP-Seq_A2780_Human
B	PI-3K cascade:FGFR2_*Homo sapiens*_R-HSA-5654695	Ionotropic glutamate receptor pathway_*Homo sapiens*_P00037	Breast cancer	Protein phosphorylation	Motor activity	AR_22383394_ChIP-Seq_PROSTATE_CANCER_Human
B	PI-3K cascade:FGFR3_*Homo sapiens*_R-HSA-5654710	Wnt signaling pathway_*Homo sapiens*_P00057	Pathways in cancer	Calcium ion transport	Voltage-gated cation channel activity	PAX3-FKHR_20663909_ChIP-Seq_RHABDOMYOSARCOMA_Human
C	PI-3K cascade:FGFR1_*Homo sapiens*_R-HSA-5654689	p53 pathway feedback loops 2_*Homo sapiens*_P04398	Endometrial cancer	protein phosphorylation (GO:0006468)	MAP kinase kinase activity	STAT3_23295773_ChIP-Seq_U87_Human
C	PI-3K cascade:FGFR2_*Homo sapiens*_R-HSA-5654695	EGF receptor signaling pathway_*Homo sapiens*_P00018	Gastric cancer	Protein autophosphorylation (GO:0046777)	Calcium ion transmembrane transporter activity	TCF4_23295773_ChIP-Seq_U87_Human
C	PI-3K cascade:FGFR3_*Homo sapiens*_R-HSA-5654710	Endothelin signaling pathway_*Homo sapiens*_P00019	Thyroid hormone signaling pathway	Calcium ion import (GO:0070509)	Protein kinase activity (GO:0004672)	SMAD4_21799915_ChIP-Seq_A2780_Human
C	PI-3K cascade:FGFR4_*Homo sapiens*_R-HSA-5654720	p53 pathway_*Homo sapiens*_P00059	Central carbon metabolism in cancer	Peptidyl-serine phosphorylation (GO:0018105)	ATPase activity (GO:0016887)	AR_22383394_ChIP-Seq_PROSTATE_CANCER_Human
C	PI3K events in ERBB4 signaling_*Homo sapiens*_R-HSA-1250342	Wnt signaling pathway_*Homo sapiens*_P00057	Breast cancer	Phosphorylation (GO:0016310)	ATP-dependent microtubule motor activity, minus-end-directed (GO:0008569)	DROSHA_22980978_ChIP-Seq_HELA_Human
D	Chromatin modifying enzymes_*Homo sapiens*_R-HSA-3247509	CCKR signaling map ST_*Homo sapiens*_P06959	Endometrial cancer	Regulation of megakaryocyte differentiation (GO:0045652)	ATP-dependent microtubule motor activity, minus-end-directed (GO:0008569)	AR_19668381_ChIP-Seq_PC3_Human
D	Chromatin organization_*Homo sapiens*_R-HSA-4839726	Wnt signaling pathway_*Homo sapiens*_P00057	Human papillomavirus infection	Regulation of myeloid cell differentiation (GO:0045637)	ATP-dependent microtubule motor activity (GO:1990939)	TCF4_23295773_ChIP-Seq_U87_Human
D	Developmental Biology_*Homo sapiens*_R-HSA-1266738	Huntington disease_*Homo sapiens*_P00029	Hepatocellular carcinoma	Cellular response to caffeine (GO:0071313)	Ligand-gated calcium channel activity (GO:0099604)	SMAD4_21799915_ChIP-Seq_A2780_Human
D	PI3K/AKT Signaling in Cancer_*Homo sapiens*_R-HSA-2219528	p53 pathway_*Homo sapiens*_P00059	Lysine degradation	Response to caffeine (GO:0031000)	Protein kinase binding (GO:0019901)	STAT3_23295773_ChIP-Seq_U87_Human
D	PKMTs methylate histone lysines_*Homo sapiens*_R-HSA-3214841	Beta1 adrenergic receptor signaling	Huntington disease	Regulation of cardiac muscle cell contraction (GO:0086004)	ATPase activity (GO:0016887)	ZNF217_24962896_ChIP-Seq_MCF-7_Human

The GSEA results suggested that our candidate genes are most significantly overrepresented in gene sets associated with pathways/biological functions contributing to breast cancer. For instance, gene sets related to pathways such as PIK3 cascade (“PI-3K cascade: FGFR1”, “PI-3K cascade: FGFR2”, “PI-3K cascade: FGFR3”, “PI3K events in ERBB4”, “PI3K/AKT Signaling in Cancer”) and TP53 pathway (“p53 pathway feedback loops 2”, “p53 pathway”) frequently appeared among the most significant results. Interestingly, consistent with the purpose of our analysis, the results from “KEGG 2019” database identified breast cancer as one of the cancers most significantly associated with the input gene lists. Accordingly, the GSEA results fully support the association between the input gene lists (genes identified as significant through our analysis) and breast cancer development.

## 4 Conclusion

Computational models for classifying the pathogenic status of cancer somatic variants located in coding and noncoding regions of the genome were developed in this study. The novelty of the proposed classification models is the development of a bi-dimensional threshold that spans the recurrent frequency of a given somatic SNV across the whole dataset as well as the number of cancer types the SNV is identified in. Furthermore, both the pathogenic somatic SNVs and benign negative somatic SNVs included in the gold standard datasets are exclusively cancer somatic variants distinguishing our models from the currently available classifiers.

The developed models outperform the most powerful available classification tools which is evidence to support the robustness of the discrimination capabilities of our models in terms of classifying cancer somatic variants. The high classification accuracy of the developed models is promising in terms of predicting the pathogenic status of a set of cancer somatic variants whose pathogenicity has not been assigned previously. The potential application of the computational models in identifying novel candidate target genes and biomarkers is also suggested through our study. Our survival analysis on pathogenic cancer somatic SNVs (predicted by our models) and their related genes highlighted the age-specific prognostic significance of a SNV and a gene impacting the survival time of young (<45 years old) breast cancer patients more than their older counterparts.

Our classification models are designed based on a robust labelling process defined for the first time in this study. However, labels from clinical wet lab experiments are assumed more reliable. To date there is no major dataset available that includes the clinical significance of cancer somatic variants based on the validated results from wet lab experiments. A higher number of features can usually positively affect the classification power of a computational model. The genomic features we used for training our models are annotations from the limited available annotating tools. A greater number of annotation tools potentially for generating more genomic features can considerably elevate the discrimination power of computational models.

As future directions, firstly, more advanced machine learning models such as deep learning could be tested to further improve the performance of the prediction systems. However, the interpretability of these advanced machine learning models might be a concern as the pathogenic prediction problem is related to patient healthcare, which requires that results are interpretable. Secondly, majority of the existing tools used for predicting pathogenic status of the SNVs were trained based on the mixture of somatic and germline pathogenic SNVs. Here we explored to train the models based on only somatic pathogenic SNVs. Although we have shown the advantages of our new tool over the two state-of-the-art existing tools, more work is still needed to compare the new tool to other existing tools. Thirdly, conducting further analysis on the prediction results from applying our models to somatic mutations from breast cancer datasets can lead to identification of novel therapeutic targets and biomarkers. Our model could also be applied to explore other cancer types, but not limited to breast cancer. Furthermore, experimental validation of the results from our model predictions can provide a strong proof of the classification performance of our computational models.

## Data Availability

The gold standard data and the source code for building the models can be found at https://github.com/qianliu1219/Cancer-somatic-SNVs-classification.
