# Supplementary material for: Computational Prediction of the Pathogenic Status of Cancer-Specific Somatic Variants
Source: Front Genet. 2022 Jan 18;12:805656. doi: 10.3389/fgene.2021.805656 (PMC8804317; doi:10.3389/fgene.2021.805656)
Supplement: Supplementary file 1 [file DataSheet1.docx]

Supplementary Material

Computational prediction of the pathogenic status of cancer-specific somatic variants

Nikta Feizi^1^, Qian Liu^2^, Leigh Murphy^1,3^, Pingzhao Hu ^1,2,3^

^1^Department of Biochemistry and Medical Genetics, University of Manitoba, Winnipeg, Manitoba, Canada

^2^Department of Computer Science, University of Manitoba, Winnipeg, Manitoba, Canada

^3^CancerCare Manitoba Research Institute, Winnipeg, Manitoba, Canada

# Supplementary Tables

**Table S1**: A summary of the features defined for the variants in coding and non-coding gold standards. Coding specific features are assigned as "Yes" in the last column

| # | Feature | Description | Feature category | Coding specific |
| --- | --- | --- | --- | --- |
| 1 | Transversion | Type of nucleotide change (Trans version/sition) | Structural & genomic context | No |
| 2 | Consequence S | Deleterious score assigned to potential impact of mutations based on VEP consequences | Structural & genomic context | No |
| 3 | Consequence category | Impact category assigned to mutations based on VEP consequences | Structural & genomic context | No |
| 4 | GC % | Percentage of GC in a +/-75 bp window | Structural & genomic context | No |
| 5 | CpG % | Percentage of CpG in a +/-75 bp window | Structural & genomic context | No |
| 6 | cDNA Pos | Distance from transcription start | Distance feature | Yes |
| 7 | Rel cDNA Pos | Relative distance from transcription start | Distance feature | Yes |
| 8 | CD start | Distance from coding start site | Distance feature | Yes |
| 9 | Rel CD start | Relative distance from coding start site | Distance feature | Yes |
| 10 | Prot Pos | Amino acid distance from coding start site | Distance feature | Yes |
| 11 | Rel Prot Pos | Relative amino acid distance from coding start site | Distance feature | Yes |
| 12 | Min dist TSS | Minimum distance to Transcribed Sequence Start(TSS) | Distance feature | No |
| 13 | Min dist TSE | Minimum distance to Transcribed Sequence End (TSE) | Distance feature | No |
| 14 | SIFT | SIFT score | Structural & genomic context | Yes |
| 15 | PolyPhen | PolyPhen score | Structural & genomic context | Yes |
| 16 | PhastCons-pri | PhastCons Primate score | Conservation | No |
| 17 | PhastCons-mam | PhastCons Mammalian score | Conservation | No |
| 18 | PhastCons-ver | PhastCons Vertebrate score | Conservation | No |
| 19 | Phylop-Pri | Phylop Primate score | Conservation | No |
| 20 | Phylop-mam | Phylop Mammalian score | Conservation | No |
| 21 | Phylop-ver | Phylop Vertebrate score | Conservation | No |
| 22 | Background S | Background selection score | Conservation | No |
| 23 | Gerp RS | Gerp RS score | Conservation | Yes |
| 24 | Gerp RS pval | Gerp RS score p-Value | Conservation | Yes |
| 25 | Gerp N | Neutral evolution score from GERP++ | Conservation | No |
| 26 | Gerp S | Rejected substitution score from GERP++ | Conservation | No |
| 27 | H3K4me1 | Maximum H3K4 methylation level from Encode | Epigenetic feature | No |
| 28 | H3K4me2 | Maximum H3K4 dimethylation level from Encode | Epigenetic feature | No |
| 29 | H3K4me3 | Maximum H3K4 trimethylation level from Encode | Epigenetic feature | No |
| 30 | H3K9ac | Maximum H3K9 acetylation level from Encode | Epigenetic feature | No |
| 31 | H3K9me3 | Maximum H3K9 trimethylation level from Encode | Epigenetic feature | No |
| 32 | H3K27ac | Maximum H3K27 acetylation level from Encode | Epigenetic feature | No |
| 33 | H3K27me3 | Maximum H3K27 trimethylation level from Encode | Epigenetic feature | No |
| 34 | H3K36me3 | Maximum H3K36 trimethylation level from Encode | Epigenetic feature | No |
| 35 | H3K79me2 | Maximum H3K79 dimethylation level from Encode | Epigenetic feature | No |
| 36 | H4K20me1 | Maximum H4K20 methylation level from Encode | Epigenetic feature | No |
| 37 | H2AFZ | Maximum level of H2A Histone Family Member Z | Epigenetic feature | No |
| 38 | DNase | Maximum DNase-seq level form Encode | Epigenetic feature | No |
| 39 | RNA | Maximum total RNA-seq level form Encode | Epigenetic feature | No |
| 40 | Grantham | Grantham score | Conservation feature | Yes |
| 41 | PHRED | CADD PHRED score | Structural & genomic context | No |
| 42 | cHMM-E1 | Number of 48 cell types in chromosome state 1 from chromHMM | Epigenetic feature | No |
| 43 | cHMM-E2 | Number of 48 cell types in chromosome state 2 from chromHMM | Epigenetic feature | No |
| 44 | cHMM-E3 | Number of 48 cell types in chromosome state 3 from chromHMM | Epigenetic feature | No |
| 45 | cHMM-E4 | Number of 48 cell types in chromosome state 4 from chromHMM | Epigenetic feature | No |
| 46 | cHMM-E5 | Number of 48 cell types in chromosome state 5 from chromHMM | Epigenetic feature | No |
| 47 | cHMM-E6 | Number of 48 cell types in chromosome state 6 from chromHMM | Epigenetic feature | No |
| 48 | cHMM-E7 | Number of 48 cell types in chromosome state 7 from chromHMM | Epigenetic feature | No |
| 49 | cHMM-E8 | Number of 48 cell types in chromosome state 8 from chromHMM | Epigenetic feature | No |
| 50 | cHMM-E9 | Number of 48 cell types in chromosome state 9 from chromHMM | Epigenetic feature | No |
| 51 | cHMM-E10 | Number of 48 cell types in chromosome state 10 from chromHMM | Epigenetic feature | No |
| 52 | cHMM-E11 | Number of 48 cell types in chromosome state 11 from chromHMM | Epigenetic feature | No |
| 53 | cHMM-E12 | Number of 48 cell types in chromosome state 12 from chromHMM | Epigenetic feature | No |
| 54 | cHMM-E13 | Number of 48 cell types in chromosome state 13 from chromHMM | Epigenetic feature | No |
| 55 | cHMM-E14 | Number of 48 cell types in chromosome state 14 from chromHMM | Epigenetic feature | No |
| 56 | cHMM-E15 | Number of 48 cell types in chromosome state 15 from chromHMM | Epigenetic feature | No |
| 57 | cHMM-E16 | Number of 48 cell types in chromosome state 16 from chromHMM | Epigenetic feature | No |
| 58 | cHMM-E17 | Number of 48 cell types in chromosome state 17 from chromHMM | Epigenetic feature | No |
| 59 | cHMM-E18 | Number of 48 cell types in chromosome state 18 from chromHMM | Epigenetic feature | No |
| 60 | cHMM-E19 | Number of 48 cell types in chromosome state 19 from chromHMM | Epigenetic feature | No |
| 61 | cHMM-E20 | Number of 48 cell types in chromosome state 20 from chromHMM | Epigenetic feature | No |
| 62 | cHMM-E21 | Number of 48 cell types in chromosome state 21 from chromHMM | Epigenetic feature | No |
| 63 | cHMM-E22 | Number of 48 cell types in chromosome state 22 from chromHMM | Epigenetic feature | No |
| 64 | cHMM-E23 | Number of 48 cell types in chromosome state 23 from chromHMM | Epigenetic feature | No |
| 65 | cHMM-E24 | Number of 48 cell types in chromosome state 24 from chromHMM | Epigenetic feature | No |
| 66 | cHMM-E25 | Number of 48 cell types in chromosome state 25 from chromHMM | Epigenetic feature | No |
| 67 | Intron-Exon | Binary variable, if the SNV is located in intron its score is 0 and if its located in exon the score is 1 | Structural & genomic context | Yes |
| 68 | Domain-VEP | Dummy variable based on Domain annotations from VEP (e.g. if the variants is located in a sigp) | Structural & genomic context | Yes |
| 69 | BRAVO-Freq100bp | Count of frequent SNVs (MAF>0.05) in 100 bp window distance from the mutation base on BRAVO | Structural & genomic context | No |
| 70 | BRAVO-Rare100bp | Count of rare SNVs (MAF<0.05) in 100 bp window distance from the mutation base on BRAVO | Structural & genomic context | No |
| 71 | BRAVO-Sngl100bp | Count of single occurrence of the SNVs (MAF<0.05) in 100 bp window distance base on BRAVO | Structural & genomic context | No |
| 72 | BRAVO-Freq1000bp | Count of frequent SNVs (MAF>0.05) in 1000 bp window distance from the mutation base on BRAVO | Structural & genomic context | No |
| 73 | BRAVO-Rare1000bp | Count of rare SNVs (MAF<0.05) in 1000 bp window distance from the mutation base on BRAVO | Structural & genomic context | No |
| 74 | BRAVO-Sngl1000bp | Count of single occurrence of the SNVs (MAF<0.05) in 100 bp window distance base on BRAVO | Structural & genomic context | No |
| 75 | BRAVO-Freq10000bp | Count of frequent SNVs (MAF>0.05) in 10000 bp window distance from the mutation base on BRAVO | Structural & genomic context | No |
| 76 | BRAVO-Rare10000bp | Count of rare SNVs (MAF<0.05) in 1000 bp window distance from the mutation base on BRAVO | Structural & genomic context | No |
| 77 | BRAVO-Sngl10000bp | Count of single occurrence of the SNVs (MAF<0.05) in 100 bp window distance base on BRAVO | Structural & genomic context | No |
| 78 | BRAVO-dist-mutation | Distance between closest up and down BRAVO SNVs | Distance feature | No |
| 79 | RemapOverlap TF | Number of different transcription factor binding affected by the mutation from Remap | Structural and genomic context | Yes |
| 80 | RemapOverlap CL | Number of different transcription factor binding – cell line combination affected by the mutation from Remap | Structural and genomic context | Yes |

Table S2: Number of positive examples per different thresholds in coding regions. The highlighted cells show the chosen threshold for each category. Our gold-standard includes the SNVs passing both filters.

| Threshold | Number of positive samples across whole dataset | Number of positive samples based on number of cancer types |
| --- | --- | --- |
| 1 | 3,569,793 | 3,318,128 |
| 2 | 798,856 | 206,349 |
| 3 | 261,833 | 30,294 |
| 4 | 122,054 | 6,999 |
| 5 | 67,028 | 2,601 |
| 6 | 42,328 | 1,484 |
| 7 | 31,437 | 977 |
| 8 | 25,624 | 674 |
| 9 | 21,739 | 494 |
| 10 | 19,238 | 372 |
| 11 | 17,299 | 309 |
| 12 | 15,816 | 249 |
| 13 | 14,671 | 191 |
| 14 | 13,868 | 141 |

Table S3: Number of positive examples per different thresholds in non-coding regions. The highlighted cells show the chosen threshold for each category. Our gold-standard includes the SNVs passing both filters.

| Threshold | Number of positive samples across whole dataset | Number of positive samples based on number of cancer types |
| --- | --- | --- |
| 1 | 16,966,056 | 16,796,255 |
| 2 | 1,405,383 | 156,816 |
| 3 | 266,704 | 11,117 |
| 4 | 115,861 | 1,507 |
| 5 | 58,561 | 266 |
| 6 | 31,454 | 49 |
| 7 | 20,039 | 9 |
| 8 | 13,897 | 4 |
| 9 | 8,760 | 2 |
| 10 | 3,875 | 2 |
| 11 | 2,464 | 2 |
| 12 | 1,701 | 2 |
| 13 | 1,198 | 2 |
| 14 | 905 | 2 |

Table S4: Coefficient of features from the Lasso model of the coding regions. The absolute value of the coefficient denotes the importance of each feature in distinguishing between pathogenic and non-pathogenic groups. The coefficient of 0 implies that the feature is not selected in the feature selection process.

| Feature | Coef | Group | Feature | Coef | Group | Feature | Coef | Group |
| --- | --- | --- | --- | --- | --- | --- | --- | --- |
| CpG | 5.430427368 | Structural | cHmm_E17 | 0.02981 | Epigenetic | Sngl100bp | -0.00439 | Structural |
| GC | -2.152262409 | Structural | cHmm_E22 | 0.028782 | Epigenetic | cHmm_E14 | -0.00437 | Epigenetic |
| Freq100bp | -2.083440449 | Structural | Freq1000bp | 0.023307 | Structural | H3K4me1 | -0.00391 | Epigenetic |
| Rare100bp | -0.700953376 | Structural | cHmm_E19 | -0.02256 | Epigenetic | cHmm_E8 | -0.00375 | Epigenetic |
| relcDNApos | -0.482798812 | Distance | H4K20me1 | 0.019239 | Epigenetic | Domain_dmm | 0.003591 | Structural |
| Transversion | -0.452752695 | Structural | cHmm_E11 | 0.018627 | Epigenetic | H3K27me3 | 0.003292 | Epigenetic |
| SIFTval | -0.444132285 | Structural | DNase | 0.018349 | Epigenetic | Sngl1000bp | 0.002419 | Structural |
| verPhCons | -0.375352798 | Conservation | cHmm_E21 | 0.017092 | Epigenetic | cHmm_E15 | -0.002 | Epigenetic |
| priPhyloP | -0.346568505 | Conservation | cHmm_E10 | -0.017 | Epigenetic | Grantham | -0.00193 | conservation |
| Int_Ex | -0.260106506 | Structural | cHmm_E25 | -0.01388 | Epigenetic | cHmm_E7 | 0.001807 | Epigenetic |
| mamPhCons | -0.22078794 | Conservation | H3K79me2 | 0.013789 | Epigenetic | totalRNA | 0.00168 | Epigenetic |
| priPhCons | -0.17883215 | Conservation | cHmm_E16 | 0.012791 | Epigenetic | cHmm_E24 | -0.00142 | Epigenetic |
| Cons_dummy | -0.124538094 | Structural | H2AFZ | 0.011427 | Epigenetic | Rare1000bp | -0.00058 | Structural |
| relCDSpos | 0.115313109 | Distance | GerpS | -0.01135 | Conservation | Rare10000bp | -0.0005 | Structural |
| PHRED | 0.112139281 | Structural | cHmm_E6 | -0.01035 | Epigenetic | OverlapCL | 0.000386 | Structural |
| PolyPhenVal | 0.110474063 | Structural | cHmm_E9 | -0.01029 | Epigenetic | Sngl10000bp | 0.000109 | Structural |
| ConsScore | 0.107390987 | Structural | H3K27ac | 0.009979 | Epigenetic | bStatistic | -5.89E-05 | Conservation |
| H3K9me3 | 0.092988449 | Epigenetic | GerpN | -0.00941 | Conservation | CDSpos | -3.06E-05 | Distance |
| verPhyloP | 0.073078108 | Conservation | H3K9ac | -0.00799 | Epigenetic | GerpRS | 2.97E-05 | Conservation |
| mamPhyloP | -0.062205422 | Conservation | H3K4me2 | -0.00697 | Epigenetic | cDNApos | 1.99E-05 | Distance |
| cHmm_E23 | -0.053494173 | Epigenetic | H3K4me3 | -0.00644 | Epigenetic | protPos | -7.00E-06 | Distance |
| cHmm_E1 | 0.04944372 | Epigenetic | Freq10000bp | -0.00613 | Structural | Dist2Mutation | 5.21E-06 | Distance |
| cHmm_E13 | 0.046496532 | Epigenetic | cHmm_E12 | 0.005691 | Epigenetic | minDistTSS | 9.78E-07 | Distance |
| cHmm_E18 | -0.040574404 | Epigenetic | cHmm_E2 | -0.00531 | Epigenetic | minDistTSE | -4.95E-07 | Distance |
| cHmm_E3 | 0.037666804 | Epigenetic | OverlapTF | -0.00487 | Structural | relProtPos | 0 | Distance |
| cHmm_E5 | 0.035345748 | Epigenetic | H3K36me3 | 0.00477 | Epigenetic | GerpRSpval | 0 | Conservation |
| cHmm_E4 | 0.031469897 | Epigenetic | cHmm_E20 | -0.00445 | Epigenetic |  |  |  |

Table S5: Coefficient of features from the Lasso model in the non-coding regions. The absolute value of the coefficient denotes the importance of each feature in distinguishing between pathogenic and non-pathogenic groups.

| Feature | Coef | Group | Feature | Coef | Group | Feature | Coef | Group |
| --- | --- | --- | --- | --- | --- | --- | --- | --- |
| CpG | 4.061880549 | Structural | cHmm_E2 | 0.019548838 | Epigenetics | cHmm_E14 | 0.005800379 | Epigenetics |
| Freq100bp | 1.690500049 | Structural | H3K4me1 | 0.017862322 | Epigenetics | H3K79me2 | 0.005246062 | Epigenetics |
| Transversion | 0.640151714 | Structural | H3K4me3 | 0.017224207 | Epigenetics | cHmm_E23 | 0.005154772 | Epigenetics |
| GC | 0.597857517 | Structural | cHmm_E25 | 0.016014631 | Epigenetics | GerpS | 0.004004753 | Conservation |
| verPhCons | 0.469939117 | Conservation | PHRED | 0.015900759 | Structural | totalRNA-max | 0.003576802 | Epigenetics |
| Rare100bp | 0.423388778 | Structural | cHmm_E10 | 0.015729031 | Epigenetics | cHmm_E8 | 0.002611941 | Epigenetics |
| ConsScore | 0.310816596 | Structural | cHmm_E12 | 0.014548169 | Epigenetics | cHmm_E7 | 0.00194307 | Epigenetics |
| priPhCons | 0.224622944 | Conservation | Freq1000bp | 0.01333975 | Structural | H3K27ac | 0.001560174 | Epigenetics |
| mamPhCons | 0.200537783 | Conservation | cHmm_E21 | 0.012206939 | Epigenetics | Sngl1000bp | 0.001515173 | Structural |
| priPhyloP | 0.187248332 | Conservation | cHmm_E9 | 0.011978767 | Epigenetics | cHmm_E16 | 0.001471679 | Epigenetics |
| Cons_dummy | 0.179209493 | Structural | cHmm_E22 | 0.011843811 | Epigenetics | cHmm_E3 | 0.001347666 | Epigenetics |
| cHmm_E1 | 0.071626125 | Epigenetics | cHmm_E15 | 0.011703168 | Epigenetics | Rare10000bp | 0.001048594 | Structural |
| H3K36me3 | 0.070601559 | Epigenetics | cHmm_E11 | 0.010543743 | Epigenetics | DNase | 0.000579038 | Epigenetics |
| mamPhyloP | 0.066234151 | Conservation | cHmm_E24 | 0.010133424 | Epigenetics | H3K4me2 | 0.000502246 | Epigenetics |
| verPhyloP | 0.056137415 | Conservation | H3K9ac | 0.009987905 | Epigenetics | cHmm_E5 | 0.00043036 | Epigenetics |
| H3K9me3 | 0.045100854 | Epigenetics | cHmm_E19 | 0.009336748 | Epigenetics | cHmm_E4 | 0.000227864 | Epigenetics |
| H2AFZ | 0.03795365 | Epigenetics | cHmm_E20 | 0.008293349 | Epigenetics | Sngl10000bp | 0.000141943 | Structural |
| cHmm_E13 | 0.024148465 | Epigenetics | Rare1000bp | 0.007715412 | Structural | bStatistic | 0.0000823 | Conservation |
| cHmm_E18 | 0.023008619 | Epigenetics | cHmm_E17 | 0.007030198 | Epigenetics | Dist2Mutation | 0.0000225 | Distance |
| H3K27me3 | 0.022805297 | Epigenetics | GerpN | 0.006913753 | Conservation | minDistTSS | 0.000000379 | Distance |
| cHmm_E6 | 0.021209513 | Epigenetics | Freq10000bp | 0.00635071 | Structural | minDistTSE | 0.000000304 | Distance |
| H4K20me1 | 0.020029121 | Epigenetics | Sngl100bp | 0.005881123 | Structural |  |  |  |

Tables S6: Summary of the model performance in the coding regions. “Norm” column indicates whether the analysis were conducted for normalized data or not (“Yes” stands for normalized and “No” stands for non-normalized data). Train/Test column shows the model evaluation strategy (10 F CV stands for 10-fold cross-validation and 2/3 stands for Training (2/3) – test (1/3) setting).

| Model | Norm | Training/Test | AUC | Accuracy |
| --- | --- | --- | --- | --- |
| Lasso | No | 10F CV | 0.88 | 0.81 |
| Lasso | No | 2/3 | 0.88 | 0.80 |
| Lasso | Yes | 10F CV | 0.88 | 0.81 |
| Lasso | Yes | 2/3 | 0.89 | 0.81 |
| SVM-“rbf” | No | 10F CV | 0.54 | 0.68 |
| SVM-“rbf” | No | 2/3 | 0.53 | 0.68 |
| SVM-“rbf” | Yes | 10F CV | 0.94 | 0.88 |
| SVM-“rbf” | Yes | 2/3 | 0.93 | 0.87 |

Table S7: Summary of the model performance in the noncoding regions. “Norm” column indicates whether the analysis was conducted for normalized data or not (“Yes” stands for normalized and “No” stands for non-normalized data). Train/Test column shows the model evaluation strategy (10 F CV stands for 10-fold cross-validation and 2/3 stands for Training (2/3) – test (1/3) setting).

| Model | Norm | Train/Test | AUC | Accuracy |
| --- | --- | --- | --- | --- |
| Lasso | No | 10F CV | 0.83 | 0.78 |
| Lasso | No | 2/3 | 0.84 | 0.78 |
| Lasso | Yes | 10F CV | 0.83 | 0.78 |
| Lasso | Yes | 2/3 | 0.83 | 0.78 |
| SVM-“rbf” | No | 10F CV | 0.54 | 0.68 |
| SVM-“rbf” | No | 2/3 | 0.53 | 0.68 |
| SVM-“rbf” | Yes | 10F CV | 0.89 | 0.88 |
| SVM-“rbf” | Yes | 2/3 | 0.89 | 0.87 |

Table S8: True positive and false positive rates of the coding region-based models at different prediction thresholds. The selected threshold yielding the highest true positive rate (0.80) is highlighted in the table.

| Threshold | True positive rate (TPR) | False positive rate (FPR) |
| --- | --- | --- |
| 0.55 | 0.80 | 0.06 |
| 0.60 | 0.78 | 0.05 |
| 0.65 | 0.75 | 0.04 |
| 0.70 | 0.73 | 0.04 |
| 0.75 | 0.70 | 0.03 |
| 0.80 | 0.65 | 0.02 |
| 0.85 | 0.60 | 0.01 |

Table S9: True positive and false positive rates of the noncoding region-based models at different prediction thresholds. The selected threshold yielding the highest true positive rate (0.80) is highlighted in the table.

| Threshold | True positive rate (TPR) | False positive rate (FPR) |
| --- | --- | --- |
| 0.41 | 0.80 | 0.09 |
| 0.45 | 0.77 | 0.08 |
| 0.50 | 0.75 | 0.07 |
| 0.55 | 0.71 | 0.06 |
| 0.60 | 0.67 | 0.05 |
| 0.65 | 0.62 | 0.04 |
| 0.70 | 0.57 | 0.03 |

Table S10: The twenty-one predicted somatic pathogenic SNVs overlapped between the TCGA and METABRIC cohorts.

| 16:67612031_C>G | 17:7670685_G>A | 17:7674200_T>A |
| --- | --- | --- |
| 17:7674221_G>A | 17:7674872_T>C | 17:7674894_G>A |
| 17:7674945_G>A | 17:7674947_A>G | 17:7674953_T>C |
| 17:7675064_G>C | 17:7675076_T>C | 17:7675088_C>T |
| 3:179199088_G>A | 3:179199156_A>G | 3:179203765_T>A |
| 3:179210192_T>C | 3:179210291_G>A | 3:179218294_G>A |
| 3:179218303_G>A | 3:179221146_G>A | 3:179234297_A>T |


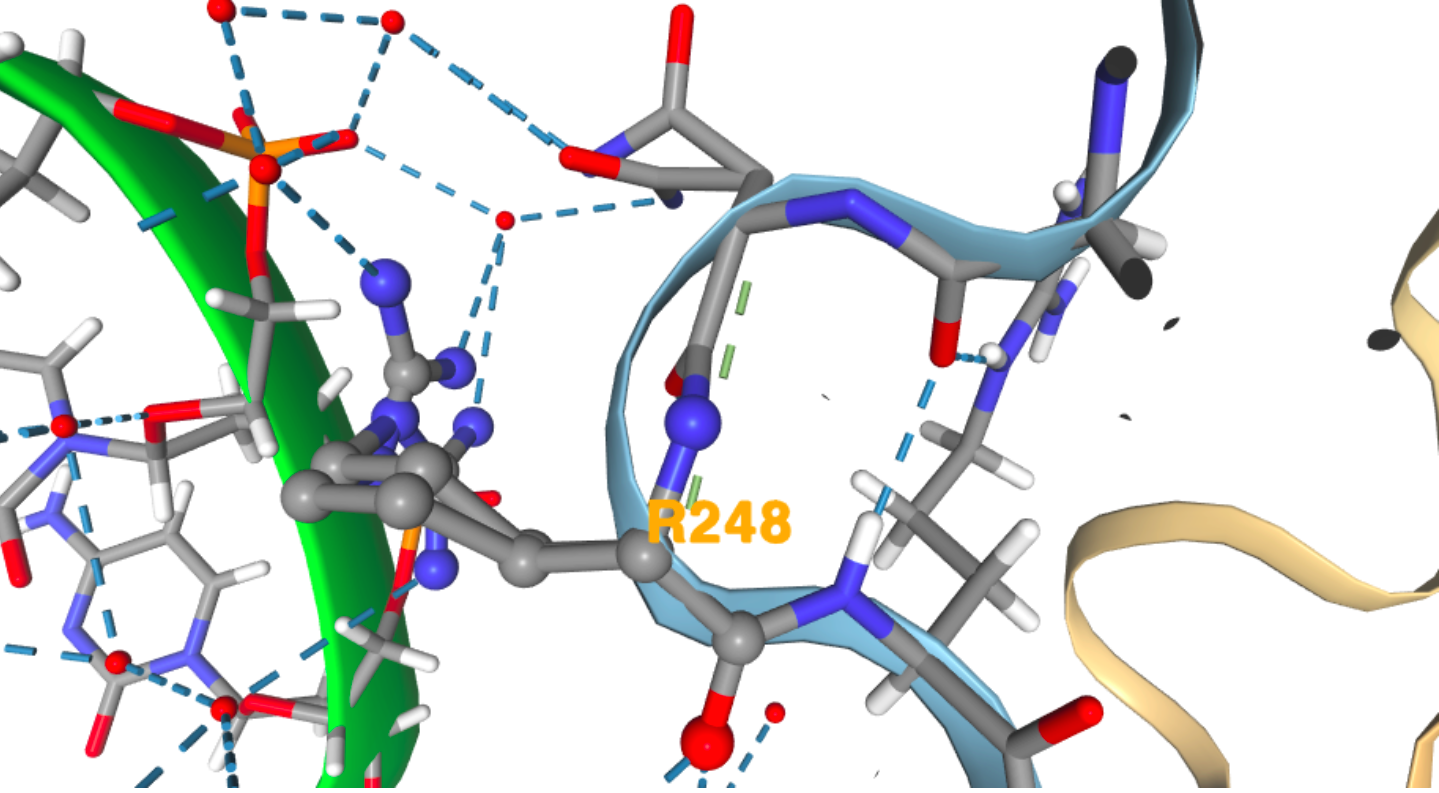


**Figure S1: The biophysical and biochemical impact of the predicted somatic pathogenic SNV prognostic 17:7674220_C>T on the 3D structure of TP53 protein.** The SNV is also named as R248. The green dash line indicates the attractive, noncovalent interaction (Pi-stacking). The blue dash line indicates the hydrogen bonds.
